# Multi-target mechanism of *Tripteryguim wilfordii* Hook for treatment of ankylosing spondylitis based on network pharmacology and molecular docking

**DOI:** 10.1080/07853890.2021.1918345

**Published:** 2021-07-14

**Authors:** Jing Zhang, Yiting Zhou, Zhiyuan Ma

**Affiliations:** a Department of Pharmacy, Sir Run Run Shaw Hospital, School of Medicine, Zhejiang University, Hangzhou, China; b Department of Clinical Pharmacology, Key Laboratory of Clinical Cancer Pharmacology and Toxicology Research of Zhejiang Province, Affiliated Hangzhou First People's Hospital, Zhejiang University School of Medicine, Hangzhou, Zhejiang, China

**Keywords:** *Tripterygium wilfordii* Hook, ankylosing spondylitis, network pharmacology, molecular docking

## Abstract

**Background:**

*Tripteryguim wilfordii* Hook (TWH) has significant anti-inflammatory and immunosuppressive properties and is widely used for treating autoimmune and inflammatory diseases. However, the multi-target mechanism of TWH on ankylosing spondylitis (AS) remains to be elucidated.

**Methods:**

Active components and their target proteins were screened from the Traditional Chinese Medicine Systems Pharmacology Database and Analysis Platform (TCMSP). Meanwhile, AS-related targets were obtained from the Genecards Database. After overlapping, the targets of TWH against AS were collected. Then protein–protein interaction (PPI) network and core targets analysis were conducted through STRING network platform and Cytoscape software. Moreover, molecular docking methods were utilized to confirm the high affinity between TWH and targets. Finally, DAVID online tool was used to perform gene ontology (GO) and Kyoto encyclopaedia of genes and genome (KEGG) pathway enrichment analysis of overlapping targets.

**Results:**

The TCMSP Database results showed that there were11 active components of TWH against AS. PPI network and core targets analysis suggested that ESR1, VEGF, ICAM-1, and RELA were key targets against AS. Moreover, molecular docking methods confirmed the high affinity between bioactive molecular of TWH and their targets in AS. At last, enrichment analysis indicated that TWH participates in various biological processes, such as cell–cell adhesion, regulation of cell–matrix adhesion, acute inflammatory response, via TNF-α, NF-κB and so forth signalling pathways.

**Conclusion:**

Verified by network pharmacology approach based on data mining and molecular docking methods, multi-target drug TWH may serve as a promising therapeutic candidate for AS but still needs further *in vivo*/*in vitro* experiments.

## Introduction

Ankylosing spondylitis (AS) is a chronic inflammatory disease that inflammation and structural damage mainly occurs in the sacroiliac joint or spine. This inflammatory damage normally causes chronic back pain and morning stiffness in patients, resulting in restriction of spinal mobility [1]. Moreover, it frequently causes aggravation in extra-articular, for instance, uveitis, psoriasis and inflammatory bowel disease [2]. More than 90% genetic role has been estimated, the strongest association being with HLA-B27 [3]. However, the detailed pathogenic role of HLA-B27 remains elusive despite the existence of multiple hypotheses.

The treatment of AS has improved significantly over the last decades with non-steroidal anti-inflammatory drugs (NSAIDs), disease-modifying antirheumatic drugs (DMARDs), TNF-specific agents and other biologics. However, in pace with long-term NSAIDs therapy, the potential risks including cardiovascular, gastrointestinal and renal related risks increase [4]. DMARDs are not recommended in the treatment of axial spondyloarthritis, since they do not affect axial spondyloarthritis only, but only a limited role for the treatment of peripheral manifestations when it coexists with the axial disease [5]. Discontinuation of TNF-blocker treatment in patients generally resulted in a relapse that occurred in 79.2% defined as responders and 84.6% of partial remission according to ASAS40 criteria [6]. Also, the high cost of biologics has always been a concern. It is noteworthy that, different from other rheumatic diseases, AS is especially characterized by the formation of bony spurs, thus control of the new bone formation remains the primary goal in the treatment of AS. The current drugs can relieve the signs and symptoms, however, fail to adequately prevent or reverse structural damage.

*Tripterygium wilfordii* Hook (TWH), a woody vine of the genus Tripterygium, is a traditional Chinese medicine with anti-inflammatory, anti-rheumatic and immunomodulatory effects. Therefore, it is widely used in a variety of autoimmune diseases, including rheumatic diseases, Crohn’s disease, systemic lupus erythematosus, and Behcet’s disease. Triptolide, a principal ingredient in TWH, could reduce collagen formation, alkaline phosphatase activity and calcium deposition *in vitro*, therefore suggests a potential therapeutic agent for the treatment of AS [7]. Additionally, comprising of triptolides, tripterygium glycosides tablet is proved to have beneficial effects in improving the clinical features and regulating serum biomarkers of patients with AS [8]. However, the mechanism underlying the therapeutic effects of TWH on AS is unknown. Based on public databases and publicly available data, network pharmacology is a novel, promising, and cost-effective approach in discovering bioactive ingredients, predicting drug action targets, and analyzing drug action mechanisms from the perspective of biological network balance [9]. Besides, compared with experimental pharmacology methods, network pharmacology emphasizes multi-channel regulation of signalling pathways, therefore especially suitable for the explanation of the mechanism of traditional Chinese medicine with multiple chemical components and molecular targets [10].

**Figure 1.**
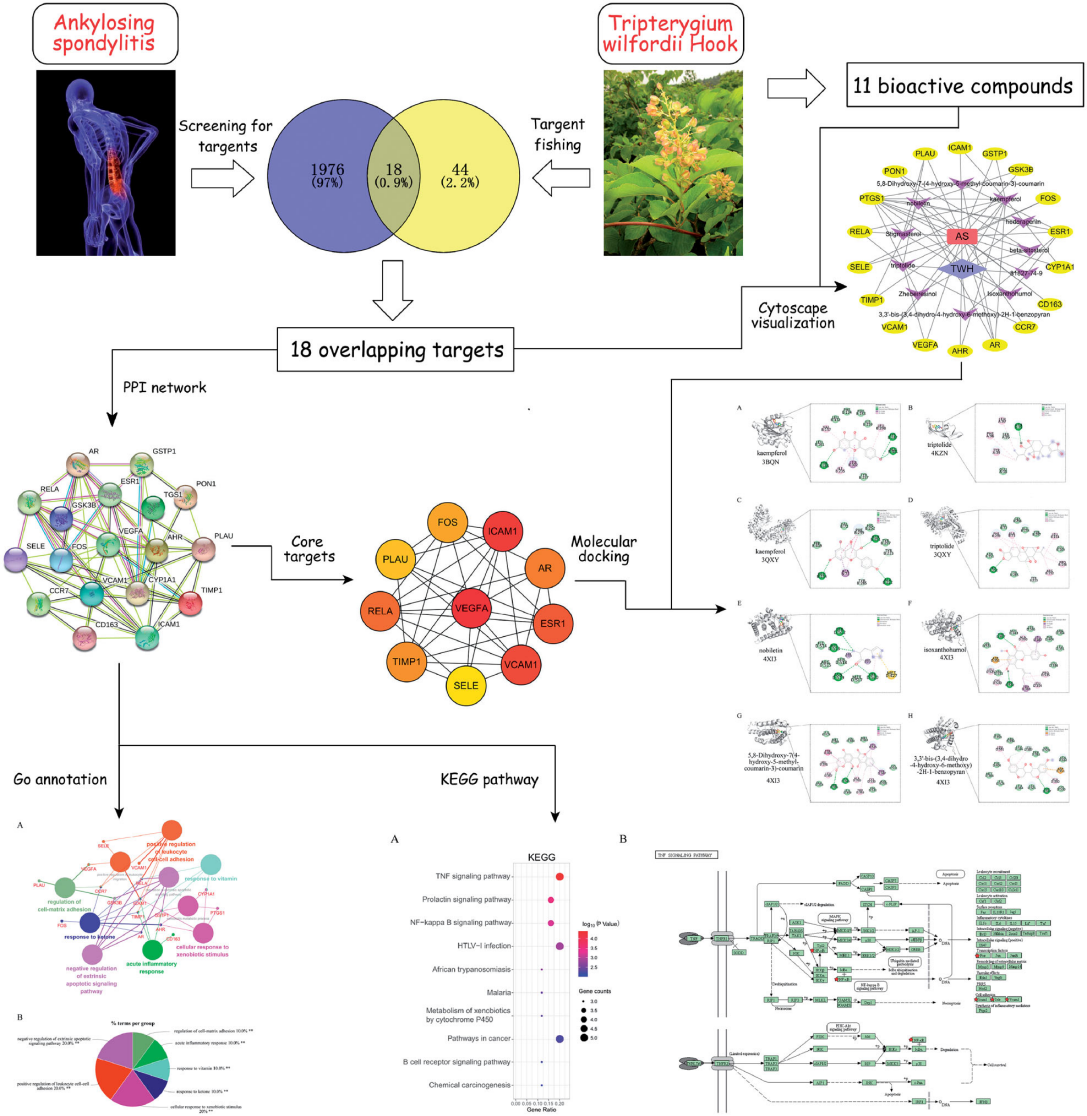
Workflow of the study design.

Herein we applied network pharmacology to analyze the active ingredients, predict core targets and pathways of TWH for treatment of AS, then preliminarily verified these targets by using molecular docking. The study design and workflow are presented in Figure 1.

## Materials and methods

### Screening for active components and target proteins

Active components of TWH were searched by Traditional Chinese Medicine Systems Pharmacology Database and Analysis Platform (TCMSP, http://tcmspw.com/tcmsp) [11]. Oral bioavailability (OB) refers to the rate and extent of a drug absorbed into the body circulation. Drug-like (DL) properties reflect the nature of a drug that has a specific functional group or contains the same or similar physical characteristics. The human intestinal cell line Caco-2 is a useful tool for studying the absorption and transport of drugs in intestinal epithelial cells. The drug half-life reflects the concentration of the drug in the blood or body and is an important parameter for calculating the dosing interval, the dose administered, and the drug accumulation [10]. The compounds with higher activity were further screened under the conditions that OB >30%, DL >0.18, Caco-2 permeability > −0.4, and half-life >3 h as reported previously [12]. Then the target proteins corresponding to each molecular were also obtained in the TCMSP Database and converted to a unified gene name using the protein database UniProt (http://www.uniprot.org/uploadlists/).

### Collection of as related targets

The Genecards Database (https://www.genecards.org/) is a platform for providing all known human genes in genome, proteome, transcription, heredity and function [13]. AS-related targets were collected from the Genecards Database. Then the AS targets retrieved in the Genecards Database and the target of TWH were mapped in Venny 2.1.0 to obtain the overlapping targets. Finally, 18 overlapping targets and 11 corresponding bioactive compounds were visualized in Cytoscape 3.7.1.

### PPI network and core targets analysis

To identify the potential hub targets of TWH and the interactions between them, the selected targets were introduced into the STRING network platform (https://string-db.org/) to construct the protein–protein interaction (PPI) network. The protein type was set to *Homo sapiens* and the confidence was set to medium (0.400). The top 10 scores of target protein in network string interactions were output, which was ranked by the MCC method using Cytohubba plug-in.

### Molecular docking

The structure of the drug small molecule (*Mol2 format) was download from the TCMSP Database. While the 3D structure of the target protein (*PDB format) was downloaded from PDB Database (https://www.rcsb.org/). Then dehydrated and removed the ligand of the active centre by PyMOL software. The target protein was hydrogenated and converted to * pdbqt format by AutoDock software, and the drug small molecule rotation bond was set up and saved in * pdbqt format. The location of the active pocket was built depends on the original ligand’s position. A grid box size of 15 × 15 × 15 points with a spacing of 1.0 Å between grid points was set to cover almost the entire favourable protein-binding site. The X, Y and Z centres were adjusted on the original ligand of different receptors. Grid box centred at (−28.574, −39.664, −30.944) Å, for the 4XIS (ESR1), grid box centred at (−18.801, 18.939, −6.819) Å, for the 3BQN (ICAM1), grid box centred at (61.672, 7.64, 61.975) Å, for the 3QXY (RELA), and grid box centred at (4.939, −4.563, 22.551) Å, for the 4KZN (VEGF). Finally, AutoDock Vina was used to docking, and search for the optimal conformation. Finally, using Discovery Studio software, the detailed docking information between a drug molecule and target was analyzed.

### GO annotation

GO enrichment could help us to understand the main action processes of the target. The ClueGO plug-in in Cytoscape was utilized to analyze the GO enrichment of the targets of TWH in treating AS. The protein type was set to *Homo sapiens* and only significant GO terms with *p <*.05 were shown [14].

### KEGG pathway

By observing the distribution of the target in the pathway, the KEGG pathway could further explain the role of the targets in metabolism, signal transduction and other processes. The targets were imported into the DAVID Database (https://david.ncifcrf.gov/) for pathway enrichment analysis, the recognition type was set to official gene symbol, the list type was set to GeneList, the species was limited to *Homo sapiens*, then the KEGG pathway information with a significant difference was searched out. And the bubble plot of pathways was drawn by R software (R 3.6.0 of Windows). The most enriched pathway was download from DAVID Database, and the enriched genes were marked with red stars.

## Results

### Screening for bioactive compounds and related targets of TWH in treatment of as

**Figure 2.**
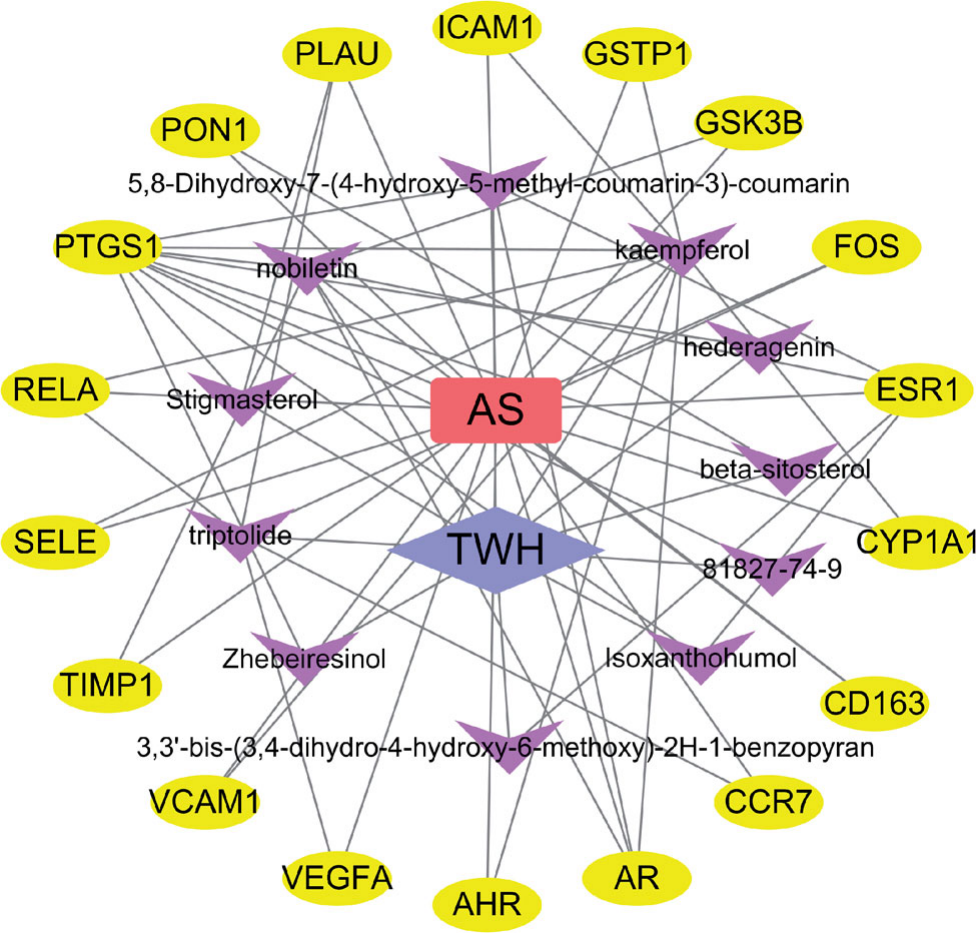
Bioactive compounds and corresponding targets network of TWH in treatment of AS.

First of all, 30 active ingredients of TWH and 62 related targets were selected through TCMSP according to the OB, DL, Caco-2 and half-life values. Next, 1994 targets of AS were obtained from Genecards Database. After mapping TWH-related targets and AS-related targets in a Venn diagram, 18 overlapping targets and corresponding target-related bioactive ingredients were accessed. Finally, 11 bioactive compounds and 18 overlapping targets which indicate targets of TWH in treatment of AS were visualized by Cytoscape (Figure 2).

### Construction of PPI network and core targets

**Figure 3.**
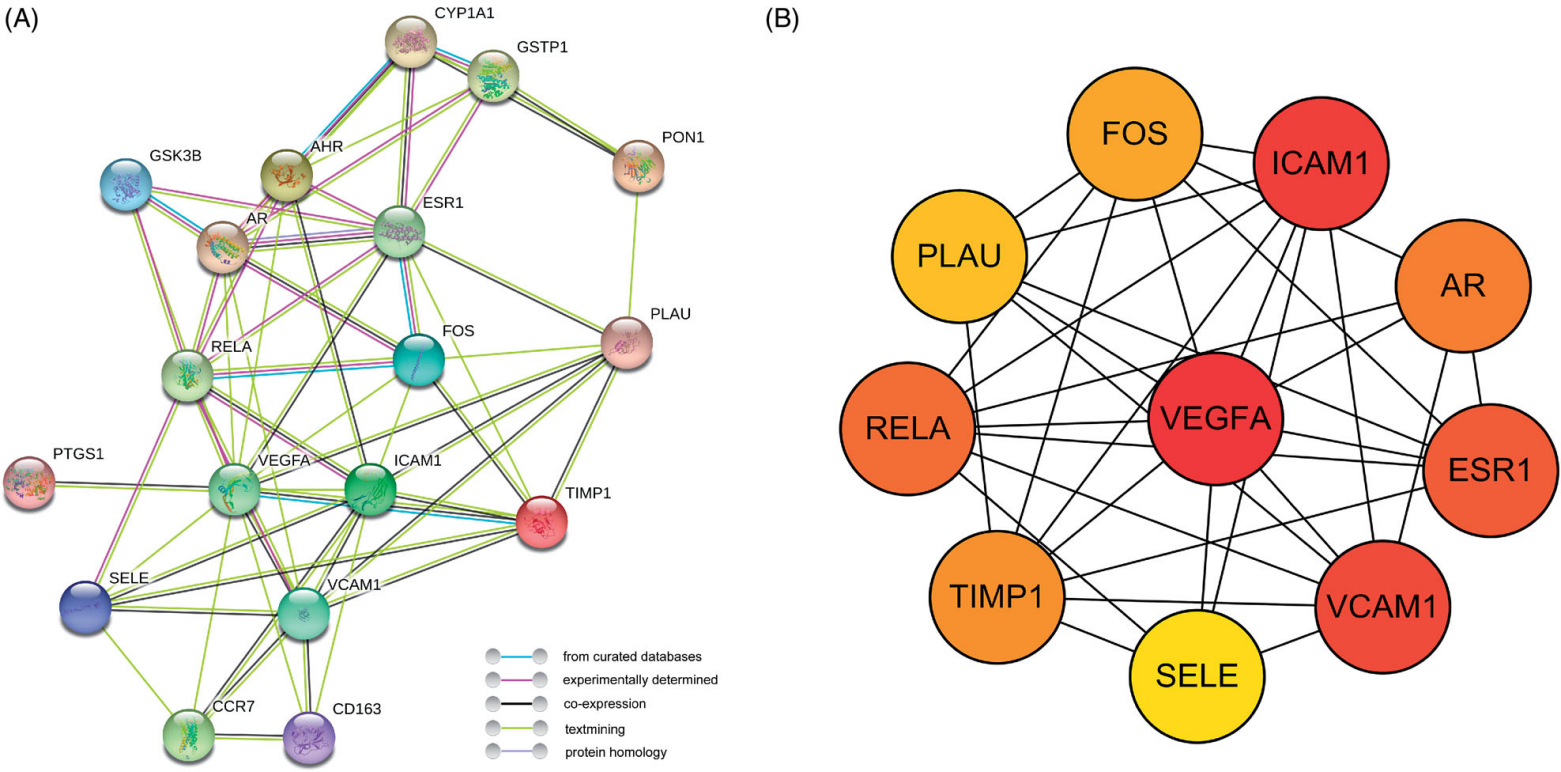
Construction of PPI network and core targets. (A) PPI network of potential targets of TWH in treatment of AS. (B) Core targets of overlapping targets.

To explore the mechanism of TWH, a PPI network was built by inputting 18 overlapping targets into the STRING Database. The target network has 18 nodes and 61 edges, with an average node degree of 6.78 (Figure 3(A)). The PPI network data built in the STRING platform was then imported into Cytoscape, and the top 10 core targets were selected by using the descending order of degree in the Cytohubba plug-in (Figure 3(B)). Due to the highest scores received, VEGFA, ICAM1, VCAM and RELA may play crucial roles in the treatment of AS.

### Validation of candidate TWH targets in treating as

**Figure 4.**
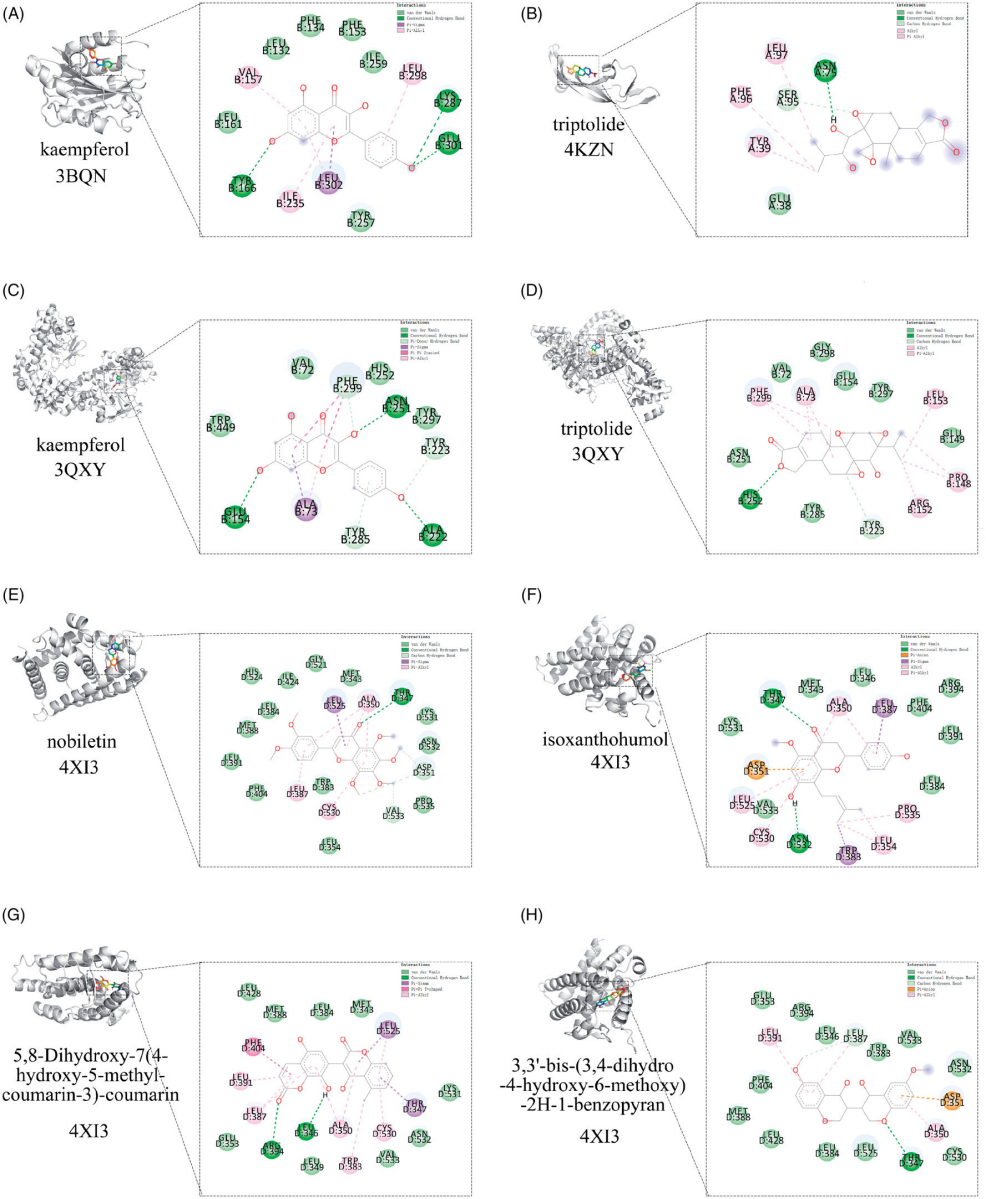
Molecular docking diagrams of AS related targets with main compounds of TWH. Kaempferol binds to protein (A) 3BQN (ICAM1) and (C) 3QXY (RELA). Triptolide is shown interacting with (B) VEGFA (4KZN) and (D) 3QXY (RELA). 4XI3 (ESR1) shows high affinity with nobiletin (E), isoxanthohumol (F), 5,8-Dihydroxy-7(4-hydroxy-5-methyl-coumarin-3)-coumarin (G), 3,3′-bis-(3,4-dihydro-4-hydroxy-6-methoxy)-2H-1-benzopyran (H).

When the drug molecular ligand binds to the target to form conformational stability, the lower the energy is, the more stable the structure is. Combining the aforementioned core targets and Cytoscape visualization results, we calculated the precision of docking between the bioactive ingredient of TWH and their potential target proteins (Table 1). Kaempferol bound to 3BQN (ICAM1) by 3 hydrogen bonds between it and TYR-166, LYS-287 and GLU-301. Other forces including van der Waals, pi–sigma, pi–alkyl bonds were also found. And it formed 3 hydrogen bonds between it and GLU-154, ALA-222, ASN-251 of 3QXY (RELA). Besides, van der Waals, pi–sigma, pi–alkyl, pi–pi stacked bonds also existed. Triptolide was attracted to 4KZN (VEGF) by 1 hydrogen bond between it and ASN-75, as well as van der Waals, alkyl, pi–alkyl bond. When encountered to 3QXY (RELA), it formed 1 hydrogen bond with HIS-252 and alkyl, pi–alkyl, van der Waals forces were also found. When the target was 4XI3 (ESR1), nobiletin, isoxanthohumol, 5,8-Dihydroxy-7(4-hydroxy-5-methyl-coumarin-3)-coumarin, 3,3′-bis-(3,4-dihydro-4-hydroxy-6-methoxy)-2H-1-benzopyran could all formed hydrogen bonds, van der Waals forces and other forces at the corresponding positions. Detailed information is depicted in Figure 4.

**Table 1.** Docking simulation for active molecular and targets of AS.

Molecular name	Targets	PBD ID	Residue involved in H bonding	H-bond length (Å)	Docking score (kcal/mol)
Kaempferol	ICAM1	3BQN	LYS287; GLU301; TYR166	1.4; 2.7; 2.9	−7.5
Triptolide	VEGF	4KZN	ASN75	2.2	−5.0
Kaempferol	RELA	3QXY	ASN251; ALA222; GLU154	3.4; 2.3; 2.8	−8.3
Triptolide	RELA	3QXY	HIS252	3.4	−8.0
Nobiletin	ESR1	4XI3	THR347	3.5	−6.6
Isoxanthohumol	ESR1	4XI3	THR347; ASN532	3.4; 2.1	−8.5
5,8-Dihydroxy-7(4-hydroxy-5-methyl-coumarin-3)-coumarin	ESR1	4XI3	LEU346; ARG394	2.6; 3.1	−8.3
3,3′-Bis-(3,4-dihydro-4-hydroxy-6-methoxy)-2H-1-benzopyran	ESR1	4XI3	THR347	3.3	−7.2

### Enrichment analysis of GO annotation and KEGG pathway

**Figure 5.**
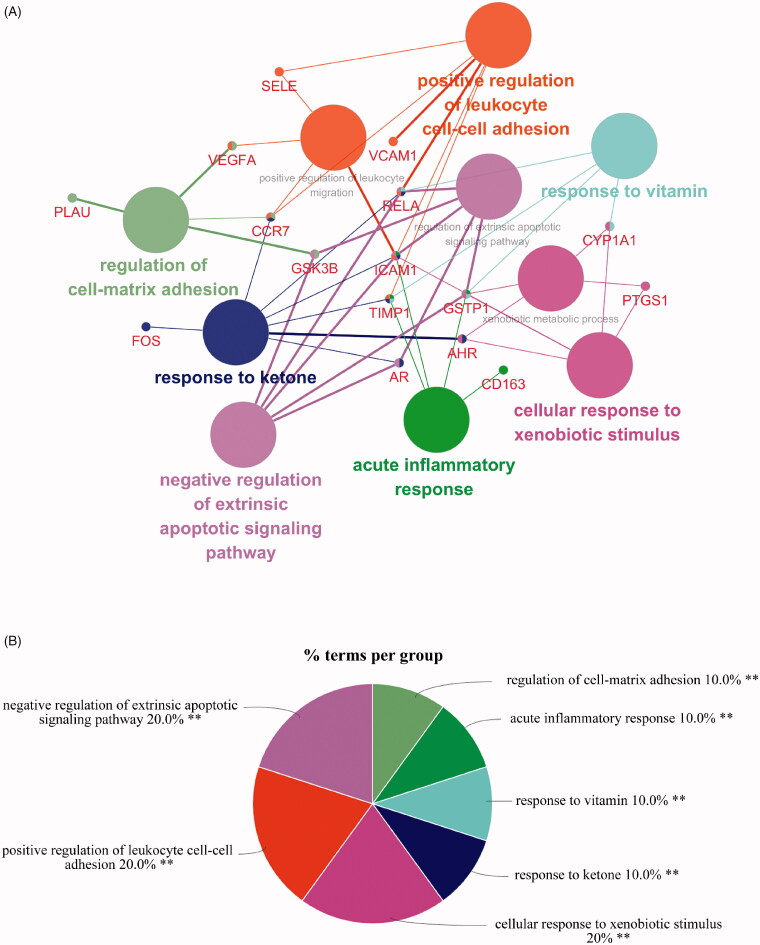
(A, B) GO enrichment analysis of key targets of TWH in treatment of AS.

**Figure 6.**
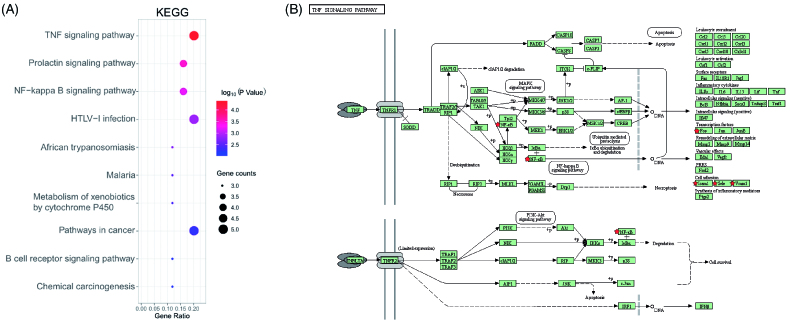
KEGG analysis of key targets of TWH in treatment of AS. (A) Bubble plot of top 10 KEGG pathways. (B) Reprinted of TNF-α signalling pathway download from DAVID Database. Gene ratio = count/set size.

Based on 18 targets of TWH obtained from the PPI network, 10 biological processes were screened out by GO biological process enrichment analysis. The visual result of enrichment analysis of GO biological process, conducted by using Cytoscape plug-in ClueGo, was shown in Figure 5. The most significantly enriched biological process were negative regulation of extrinsic apoptotic signalling pathway, positive regulation of leukocyte cell–cell adhesion, cellular response to xenobiotic stimulus, regulation of cell–matrix adhesion, acute inflammatory response, response to vitamin and response to the ketone. A total of 18 signal pathways were obtained by placing 18 genes in DAVID for KEGG pathway analysis. And top 10 enriched pathways were visualized in a bubble plot (Figure 6(A)) using the R program. The most significantly enriched pathways were the TNF signalling pathway (Figure 6(B)), prolactin signalling pathway, NF-κB signalling pathway, HTLV-1 infection, pathways in cancer, etc.

## Discussion

Unlike its rheumatologic cousin rheumatoid arthritis, AS is a common autoimmune disease characterized by both inflammation and new bone formation, however, the pathogenesis of AS has not been fully elucidated to date [15]. Thus, it is very urgent to discover safe and effective anti-AS drugs. TWH has significant anti-inflammatory and immunosuppressive properties and is widely used for treating autoimmune and inflammatory diseases, especially in traditional Chinese medicine [16]. In spite of research decipher the active ingredients and molecular mechanisms of TWH against rheumatoid arthritis, few studies explored TWH in treatment for AS with the same rheumatic condition based on network pharmacology. In this work, we employed network pharmacology and molecular docking method to clarify the molecular biological mechanism of TWH for the treatment of AS.

Angiogenesis plays important role in the pathogenesis of chronic inflammatory diseases and inflammation itself may up-regulate the expression of VEGF in rheumatic diseases [17]. In addition, VEGF plays an important role in bone formation and the disease status of AS appears to be associated with elevated VEGF levels in plasma [18]. Thus, VEGF may become a potential therapeutic intervention in AS. In our study, VEGF scored 259 and ranked first among all candidate targets by the MCC algorithm method. We predicted that VEGF was affected by triptolide based on information from publicly available databases, and supported the interactions between triptolide and VEGF through molecular docking. Our results presented that VEGF was a key target for TWH when treating AS. Surprisingly, these results are consistent with a clinical study showing that a significant reduction in the VEGF expression was found in AS patients compared with those of the controls, after 12 weeks of tripterygium glycosides tablet treatment [8].

It was reported that higher levels of ICAM-1 and VCAM-1, two adhesion molecules, were associated with impaired endothelial dysfunction in active AS [19]. Interestingly, kaempferol treatment in atherosclerosis rabbits displayed a remarkable reduction in both the gene and protein expression of ICAM-1, VCAM-1 [20]. Besides, Fang Lin et al. revealed that kaempferol could strengthen the suppression function of Treg cells and prevent the pathological symptom of collagen-induced arthritis in a rat model [21]. Briefly, all these findings provided evidence that kaempferol, an active ingredient of TWH, is sufficient to alleviate endothelial impairment and symptoms of rheumatic disease by down regulating ICAM-1and VCAM-1.

Additionally, RELA and ESR may also be key targets in AS treatment based on our calculation as well as existing reports. RELA belongs to a family of transcription factors NF-κB (nuclear factor kappa from B cells) complex that plays a fundamental role in inflammatory and immune responses. Besides damage of sacroiliac joint or spine, patients with AS frequently have decreased pulmonary function negatively correlated with the NF-κB signalling pathway, oxidative indexes, and inflammatory factors [22]. Furthermore, triptolide could protect bone loss by increasing osteoprotegerin expression by reducing the expression of the receptor for activation of NF-κB ligand (RANKL) in rats [23]. Anterior uveitis associated with AS preferentially occurs in adult men, and oestrogen receptor antagonists could down-regulate inflammatory genes in endotoxin-induced uveitis, which suggested a beneficial role of ESR in inflammatory disease [24].

KEGG analysis suggested that the TNF signalling pathway was the highest enrichment pathway. Consistent with our results, celastrol, a bioactive ingredient of TWH, could reduce active oxygen free radicals, and partially decrease the secretion of TNF-α [25]. Likewise, rats treated with triptolide were able to down-regulate the mRNA expression levels of TNF-α, thus suppressing the inflammation state in an AS spinal arthritis model [26]. Besides NF-κB and MAPK, we also performed the docking of ASK1, another TNF downstream signal, with active constituents of TWH. However, the results showed that none of the binding energy was lower than −5 kcal/mol, which indicated that ASK1 may not be a candidate target for TWH in the treatment of AS.

Although TWH has significant clinical efficacy, its toxic effects which are mainly associated with damaging the liver, spleen, kidneys, and blood circulatory system cannot be ignored [16]. Therefore, many researchers focus on how to reduce its toxicity thus enhance its curative effect. In recent years, novel dosage forms were developed. For example, conjugated triptolide, the main ingredient of TWH, to a carboxylmethyl chitosan, not only enhanced the aqueous solubility of free triptolide but also reduced drug toxicity *in vitro* and in mice model of rheumatoid arthritis [27]. And a novel nano-drug carrier system containing triptolide using poly-γ-glutamic acid-grafted di-tert-butyl L-aspartate hydrochloride was developed. This newly formed nanoparticle proved to be able to reduce the toxicity at the liver and spleen induced by triptolide, meanwhile, guaranteeing the efficacy of the drug in mice model of rheumatoid arthritis [28].
